# Cerebellar‐Cortical Connectivity and Receptor‐Specific Modulation in Older Adults Learning a Foreign Language

**DOI:** 10.1002/alz70860_105416

**Published:** 2025-12-23

**Authors:** Giovanna Bubbico, Federica Tomaiuolo, Carlo Sestieri, Golnoush Akhlaghipour, Alberto Granzotto, Antonio Ferretti, Mauro Gianni Perrucci, Stefano L. Sensi, Stefano Delli Pizzi

**Affiliations:** ^1^ Department of Neuroscience, Imaging and Clinical Sciences, “G. d’Annunzio” University of Chieti‐Pescara, Chieti, CHIETI, Italy; ^2^ Institute for Advanced Biomedical Technologies (ITAB), “G. d’Annunzio” University of Chieti‐Pescara, Chieti, Chieti, Italy; ^3^ Department of Neuroscience, Imaging and Clinical Sciences, “G. d’Annunzio” University of Chieti‐Pescara, Chieti, Chieti, Italy; ^4^ Department of Neurology, Montefiore Einstein Saul R., Korey Bronx, NY, US, NY, USA; ^5^ University of California, Irvine, Irvine, CA, USA; ^6^ Centre for Advanced Studies and Technology (CAST), “G. d’Annunzio” University of Chieti‐Pescara, Chieti, Chieti, Italy; ^7^ Department of Neuroscience, Imaging and clinical sciences, University G. d'Annunzio, Chieti, Italy; ^8^ University G. d'Annunzio, chieti, Italy; ^9^ Neurology Unit, Saint Annunziata Hospital, Chieti, Chieti, Chieti, Italy; ^10^ G. d'Annunzio University, Chieti, Chieti, Italy; ^11^ ITAB Institute of Neuroscience, Imaging and Clinical Sciences, Chieti, Chieti, Italy

## Abstract

**Background:**

Foreign language learning (FLL) in older adults is a cognitive training tool that enhances neuroplasticity and supports cognitive reserve. While its impact on cortical networks is well‐documented, little is known about its effects on subcortical structures, particularly the cerebellum. Given its role in learning and aging, we hypothesize that FLL selectively modulates cerebellar‐cortical connectivity, with changes in connectivity overlapping with regions rich in CB1 and GABAa receptor expression.

**Methods:**

We conducted a resting‐state functional connectivity (rs‐FC) MRI study on 27 older adults randomly assigned to either a four‐month FLL intervention (*n* = 14) or a control group (*n* = 13). We performed seed‐based analyses of cerebellar subregions using voxel‐wise comparisons to identify FLL‐induced connectivity changes. Additionally, we examined spatial correlations between these changes and receptor expression maps from publicly available databases.

**Results:**

The FLL group exhibited significant alterations in cerebellar‐cortical connectivity. Specifically, Crus I showed decreased connectivity with the posterior cingulate cortex, while Lobule VI exhibited increased connectivity with the superior frontal cortex. Additionally, Vermis IX showed reduced connectivity with the occipital cortex. These connectivity changes spatially overlapped with the distribution of CB1 receptors in Lobule VI and GABAa receptors in Vermis IV‐V.

**Conclusion:**

Our findings highlight the role of the cerebellum in the neural adaptations associated with FLL in older adults. The observed connectivity changes suggest a link between FLL‐induced neuroplasticity and the distribution of CB1 and GABAa receptors, shedding light on potential molecular mechanisms underlying cognitive resilience in aging.

